# ISMap*02* element targeted nested polymerase chain in the detection of *Mycobacterium avium* subsp. *paratuberculosis* in fecal samples of cattle and buffaloes

**DOI:** 10.14202/vetworld.2018.397-401

**Published:** 2018-03-31

**Authors:** Mamta Rani, Deepti Narang, Dinesh Kumar, Mudit Chandra, Sikh Tejinder Singh, G. Filia

**Affiliations:** 1 Department of Veterinary Microbiology, College of Veterinary Science, Guru Angad Dev Veterinary and Animal Sciences University, Ludhiana, Punjab, India; 2 Directorate of Livestock Farms, College of Veterinary Science, Guru Angad Dev Veterinary and Animal Sciences University, Ludhiana, Punjab, India; 3 Animal Disease Research Centre, College of Veterinary Science, Guru Angad Dev Veterinary and Animal Sciences University, Ludhiana, Punjab, India

**Keywords:** ISMap*02*, *Mycobacterium avium* subsp. *paratuberculosis*, nested polymerase chain reaction, paratuberculosis

## Abstract

**Background and Aim:**

Johne’s disease is chronic granulomatous enteritis which affects ruminants. There are many diagnostic approaches for the detection of *Mycobacterium avium* subsp. *paratuberculosis* (MAP) of which molecular detection methods using various elements are less time consuming and more accurate. The present study was conducted using ISMap*02* element for nested polymerase chain reaction (nPCR) based detection of MAP in fecal samples. The aim was to test the sensitivity and specificity of the ISMap*02* element and also to use this element for the detection of MAP in fecal samples of cattle and buffaloes.

**Materials and Methods:**

A total of 211 fecal samples of cattle and buffaloes from different herds around Ludhiana aged between 2 and 13 years were collected, and DNA extraction was done from these samples. The nPCR was carried out for the detection of MAP in fecal samples.

**Results:**

The ISMap*02* element was specific for the detection of MAP only and showed a sensitivity of detection of 7.6 fg/µL of the standard genomic DNA. Among the 211 fecal samples of cattle and buffaloes tested for the ISMap*02* element, 18 samples (8.5%) were positive for MAP.

**Conclusion:**

The ISMap*02* element is specific and sensitive for the detection of MAP in various samples, and when used in nPCR format, it can increase the sensitivity of detection.

## Introduction

Johne’s disease caused by *Mycobacterium avium* subsp*. paratuberculosis* (MAP), characterized by chronic granulomatous enteritis affecting primarily ruminants and many other species [1], manifested by persistent diarrhea, weight loss and a protein-losing enteropathy, followed eventually by death [2]. MAP has a global distribution and causes serious economic losses to the cattle industry [3]. Clinical onset of paratuberculosis is noted after 2 or more years of the initial infection, which usually occurs shortly after birth. Animals with no apparent clinical signs continue to shed the bacteria in their feces and milk [4]. The young animals are highly susceptible to MAP infection and can be easily infected as they ingest the bacteria through milk, colostrum, and the surface of the teats, which get contaminated through the cattle shed components [5,6]. Intra-uterine transmission can also occur, and MAP can also be detected in the saliva of cows, indicating this as a potential further mode of transmission [7]. Molecular methods, such as polymerase chain reaction (PCR), have been developed for the detection of MAP infection in animals [8].

The escalation of disease incidence in some areas of the world has led to the implementation of national control programs [9]. Although MAP belongs to three different strains designated “Sheep-type” (type S), “Cattle-type” (type C), and “Bison-type” (type B), there is no host specificity of these strains [10]. Complete eradication of the disease from cattle, although desirable, is extremely difficult [11]. Vaccination can be effective to control clinical disease, reducing fecal shedding and increasing productivity but it does not eliminate infection [12,13]. Different methods used in various formats of diagnosis of the MAP infection such as real-time and nested PCR (nPCR) along with immunomagnetic bead separation of the microorganism and dot blot hybridization of PCR products have made it possible to perform semiquantitative analysis [14,15]. A wide range of serological tests such as enzyme-linked immune sorbent assay (ELISA), agar gel immunodiffusion, delayed type hypersensitivity, interferon-gamma assay, fluorescence antibody test, and complement fixation test has been successfully used for the detection of MAP infection [16].

Advancements have been made in the recent years for the improvement of methods of detection of MAP DNA. *IS*900 element, which is present at multiple copies in the genome and thought to be unique to MAP [17]; however, recent studies have reported the presence of *IS900*-like sequences in non-MAP mycobacteria, which could lower the specificity of *IS900*-based assays. Therefore, new targets such as ISMav2 and ISMap*02* specific to MAP and also present as multiple copies in the genome are being used for the diagnosis of infection. ISMAP*02*-targeted nPCR is usually combined with *IS900*-targeted real-time PCR to improve detection rates [18].

The study aimed to use ISMap*02* element targeted nPCR in the detection of MAP in the fecal samples of cattle and buffaloes.

## Materials and Methods

### Ethical approval

The study was carried forward after obtaining approval from the concerned Ethical Committee (IAEC/2015/30-63).

### Animals

The study was carried out by collection of fecal samples (n=211) from cattle (n=86) and buffaloes (n=125) with a history of chronic intermittent diarrhea. The fecal samples were collected from various organized and unorganized dairy farms in and around Ludhiana, as well as from Teaching Veterinary Clinical Complex, GADVASU, Ludhiana. All the animals chosen were in the age group of 2-13 years. The samples were kept under refrigeration until further processing was done.

### DNA extraction from fecal samples

DNA extraction was carried out as per the QIAamp Fast DNA Stool Mini kit (Qiagen) with minor modifications. Fecal sample (approximately 220 mg) was taken in 2 mL microcentrifuge tube which was then placed in ice bath for 10-15 min. 1.4 mL of Inhibit X buffer was added to each sample. The samples were vortexed for 1 min for proper homogenization and were then incubated at 95°C for 5-7 min and then vortexed again for 15 s. The samples were centrifuged for 1 min at 14,000 rpm to pellet the fecal coarse materials. The supernatant was collected in a new 1.5 mL microcentrifuge tube and centrifuged at 14,000 rpm for 1 min. Meanwhile, 15 µL of proteinase K (20 mg/mL) was pipetted into a new tube. Then, 200 µL of the supernatant from the first tube was added to the tube containing proteinase K. 200 µL of buffer AL was added to the tube and vortexed for 15 s. Then, it was incubated at 70°C for 10 min. 200 µL of ethanol (96-100%) was added to this and vortexed for 15 s. The lysate was then loaded onto spin column and centrifuged for 1 min at 14,000 rpm. The filtrate was discarded, and 500 µL of buffer AW1 was added and centrifuged for 1 min at 14,000 rpm (first washing). The flow-through was discarded, and second washing was done by adding buffer AW2 (500 µL) and centrifuged for 3 min at 14,000 rpm. Flow through was discarded and after replacing with a new 2 mL collection tube, an empty spin was given to remove any residual ethanol present in the spin membrane. The column was transferred to a new labeled 1.5 mL eppendorf and the elution of the sample DNA was done by adding 15 µL elution buffer ATE directly to the QIAamp membrane, incubated for 5-10 min at room temperature for the maximum DNA elution and centrifuged at 14,000 rpm for 1 min. The elution step was repeated twice and a final elute volume of 30 µL was obtained. The eluted DNA was stored at −20°C for further use.

### nPCR analysis

Primers specific for the ISMap*02* genetic element were selected for use in a nPCR format [19]. The primer sequences for the initial amplification were 5’-GCACGGTTTTTCGGATAACGAG-3’ (forward primer) and 5’-TCAACTGCGTCACGGTGTCCT G-3’ (reverse primer) and resulted in a 278-bp product. The primers nested within the first set, 5’-GGATAACGAGACCGTGGATGC-3’ (forward primer) and 5’ -AACCGACGCCGCCAATACG-3’ (reverse primer), were used for a second amplification reaction and yielded a 117-bp product. The reaction mixture consisted of ultrapure nuclease-free water, GoTaq^®^ 2X Green Master Mix (Promega), primers (forward and reverse). Controls consisted of reaction mixture alone (negative control) and a positive control containing 1 µL of genomic DNA from MAP.

The thermocycling protocol was as follows: 1 cycle at 94°C for 5 min and 30 cycles at 94°C for 45 s, 60°C for 1 min, and 72°C for 2 min followed by a final extension cycle at 72°C for 7 min. For the nPCR, the following protocol was used with 1 µL of the amplicon from the first PCR used as the template for the second amplification: 1 cycle at 94°C for 5 min and 30 cycles at 94°C for 45 s, 60°C for 1 min, and 72°C for 2 min, followed by a final extension cycle at 72°C for 7 min. PCR amplicons were then subjected to agarose gel electrophoresis in 1× TBE, and the gels were visualized under the gel documentation system.

## Results

### Specificity assay of the ISMap*02* targeted nPCR

**Figure-1:**
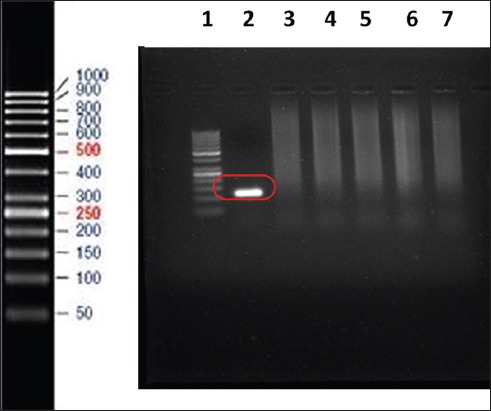
Specificity analysis of the ISMap*02* element by a conventional nested polymerase chain reaction protocol for amplification of a 117-bp product from DNA isolated from bacterial cultures of various species of mycobacteria and two non-mycobacterial species. Lane 1, 50-bp molecular mass marker; lane 2 *Mycobacterium avium* subsp. *paratuberculosi*s; lane 3 *Mycobacterium bovis*; lane 4 *Mycobacterium tuberculosis*; lanes 5 *Mycobacterium smegmatis*; lane 6 *E. coli*; lane 7 *Pasteurella multocida*.

The ISMap*02* targeted nPCR (Figure-1) was specific for MAP. The primers were tested against DNA extracted from MAP, *Mycobacterium bovis*, *Mycobacterium tuberculosis*, *Mycobacterium smegmatis*, *Escherichia coli*, and *Pasteurella multocida* organisms. None of the organisms gave products with the primers tested, except for MAP which gave a product size of 117 bp.

### Sensitivity assay of the ISMap*02* targeted nPCR

**Figure-2:**
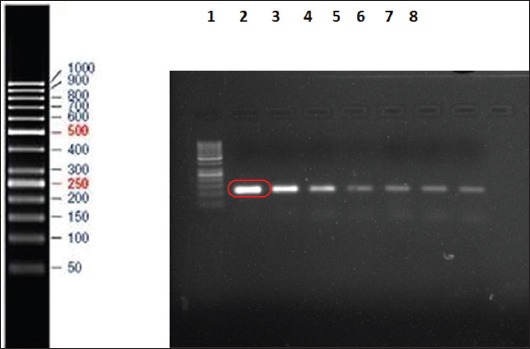
Sensitivity assay for the ISMap*02* nested polymerase chain reaction giving a product size of 117 bp. Lane 1 is 50 bp DNA marker and lane 2-8 consist of 1:10 serial dilutions of the standard *Mycobacterium avium* subsp. *paratuberculosis* genomic DNA.

The sensitivity of the primers for the ISMap*02* element was assessed by making 10-fold serial dilutions of the standard MAP genomic DNA. The initial concentration of the standard genomic DNA was checked by the nano-drop and was found to be 7.6 ng/µL. The ISMap*02* element could detect up to 7.6 fg/µL of the standard genomic DNA as shown in Figure-2.

### ISMap02 targeted nPCR

**Figure-3:**
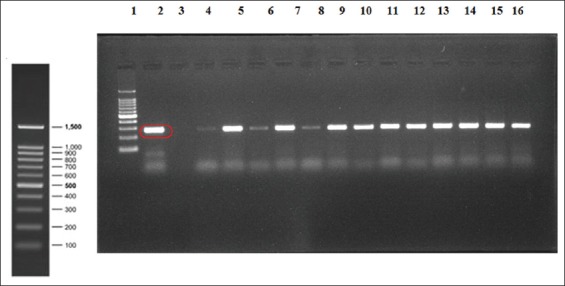
Fecal samples positive for ISMAP*02* primary product (278bp). Lane 1 consists of 100 bp DNA marker, lane 2 is positive standard genomic DNA, lane 3 is negative control, and lanes 4-16 consist of positive fecal samples for ISMap*02* primary polymerase chain reaction.

**Figure-4:**
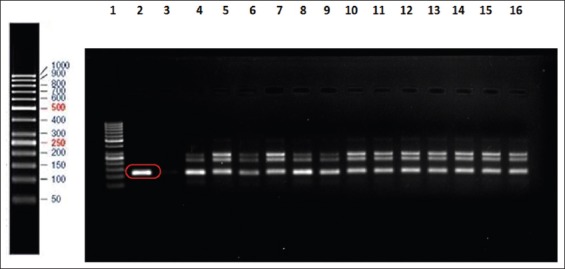
Fecal samples positive for ISMAP*02* nested product (117bp). Lane 1 consists of 50 bp DNA marker, lane 2 is positive standard genomic DNA, lane 3 is negative control, and lanes 4-16 consist of positive fecal samples for ISMap*02* nested polymerase chain reaction.

nPCR targeting the ISMap*02* element was carried out on the DNA extracted from a total of 211 fecal samples. The ISMap*02* target was detected in 18 of the 211 fecal samples representing 8.5% of the total fecal samples. The positive samples included 11 cattle and 7 buffaloes. All of these fecal samples were positive in both the rounds of the nPCR. The primary PCR product of 278bp was observed in all the 18 samples, and the nPCR product of 117bp was also seen in all the 18 samples as shown in Figures-3 and 4. In addition, two additional bands were also observed in the second round of PCR.

## Discussion

Methods of bacterial DNA detection in fecal samples have great sensitivity; therefore, PCR based detection has created a paradigm shift in the diagnostic microbiology. The methods of DNA extraction from fecal samples have become simpler and also sensitivities of the conventional PCRs are being continually improved.

In the present study, the specificity of ISMap*02* element was tested, and it was seen to be only specific for MAP DNA and not seen in *M. bovis*, *M. tuberculosis*, *M. smegmatis*, *E. coli*, and *P. multocida* organisms. Stabel and Bannantine [18] evaluated the specificity of the ISMap*02* element by PCR of the DNA extracted from isolates of MAP and *M. avium* subsp. *avium*, as well as DNA from *Mycobacterium fortuitum*, *Mycobacterium scrofulaceum*, *Mycobacterium phlei*, *Mycobacterium smegmatis*, and *Mycobacterium gordonae*. Only MAP DNA was detectable after amplification with the ISMap*02* primers.

Furthermore, the sensitivity assay was done and ISMap*02* element used for the detection of MAP could detect up to 7.6pg/µL of the standard genomic DNA isolated from the feces rather than the pure bacterial isolates. Stabel and Bannantine [18] reported the sensitivity of detection for the ISMap*02* element in either a conventional ISMap*02* nPCR or a real-time PCR format to be <100 fg DNA or 10^2^ CFU/mL in serial titration curves with pure bacteria. It was also seen that the ISMap*02* element provides a very sensitive and specific alternative as a diagnostic reagent for use in PCR assays for the detection of paratuberculosis [18]. Similarly, Mobius *et al*. [20] found that the analytical sensitivity of detection of MAP by nPCR assay was 10-100 times higher than the conventional PCR format where it could detect 10 pg/µL of DNA. The study showed that single-round PCR systems are very reliable and nPCR assays were occasionally disturbed by contaminations. This was also seen in our study where two additional bands were observed in all the 18 samples in the second round of nPCR.

nPCR increases the sensitivity and specificity of detection of MAP in fecal samples. Here, two consecutive PCR reactions are run with two sets of primers, where the products of the first reaction are used as template in the second reaction. Specificity is increased as all the four primers have to match their target in the same DNA region. Sensitivity increases as new reagents are added in the second reaction [21].

The IS900 element being used for the diagnosis of MAP infections is present at 15-20 copies within the genome of MAP [19]. However, there have been doubts and raised concerns about IS900 element being unique to MAP genome. Following the discrepancies and debates related to the specificity of IS900 element in the detection of MAP infection, many other target elements were used for MAP detection with high specificities such as F57, HspX, and genome target 251 [22,23].

The chances of false negatives are possible in the fecal samples of high shedders of MAP due to the presence of various PCR inhibitors present in the fecal matter such as phytic acid, complex mucopolysaccharides, and epithelial cells [24].

## Conclusion

ISMap*02* element is present as six copies in the genome of MAP and is specific to it as seen by the specificity analysis. It can be used as a powerful tool in the detection of MAP in various samples. In this study, we studied both sensitivity and specificity of ISMap*02* element and concluded that it could be used as a tool for the diagnosis of MAP infection in a fecal sample of cattle and buffaloes.

## Authors’ Contributions

This study was a part of M.V.Sc of MR, under the guidance of DN as Major Advisor and MC as Minor Advisor. DK helped in lab work. STS and GF supplied the samples. All authors read and approved the final manuscript.
